# Liver functional assessment using time-associated change in the liver-to-spleen signal intensity ratio on enhanced magnetic resonance imaging: a retrospective study

**DOI:** 10.1186/s12893-023-02051-1

**Published:** 2023-06-27

**Authors:** Masashi Kudo, Naoto Gotohda, Motokazu Sugimoto, Shin Kobayashi, Masaru Konishi, Tatsushi Kobayashi

**Affiliations:** 1 Department of Hepatobiliary and Pancreatic Surgery, National Cancer Center Hospital East, 6-5-1 Kashiwa-no-ha, Kashiwa, 277-8577 Chiba Japan; 2 Department of Diagnostic Radiology, National Cancer Center Hospital East, Kashiwa, Japan

**Keywords:** Dynamic, Liver function, Magnetic resonance imaging, Signal intensity, Three-dimensional

## Abstract

**Background:**

Liver-to-spleen signal intensity ratio (LSR) is evaluated by magnetic resonance imaging (MRI) in the hepatobiliary phase and has been reported as a useful radiological assessment of regional liver function. However, LSR is a passive (non-time-associated) assessment of liver function, not a dynamic (time-associated) assessment. Moreover, LSR shows limitations such as a dose bias of contrast medium and a timing bias of imaging. Previous studies have reported the advantages of time-associated liver functional assessment as a precise assessment of liver function. For instance, the indocyanine green (ICG) disappearance rate, which is calculated from serum ICG concentrations at multiple time points, reflects a precise preoperative liver function for predicting post-hepatectomy liver failure without the dose bias of ICG or the timing bias of blood sampling. The aim of this study was to develop a novel time-associated radiological liver functional assessment and verify its correlation with traditional liver functional parameters.

**Methods:**

A total of 279 pancreatic cancer patients were evaluated to clarify fundamental time-associated changes to LSR in normal liver. We defined the time-associated radiological assessment of liver function, calculated using information on LSR from four time points, as the “LSR increasing rate” (LSRi). We then investigated correlations between LSRi and previous liver functional parameters. Furthermore, we evaluated how timing bias and protocol bias affect LSRi.

**Results:**

Significant correlations were observed between LSRi and previous liver functional parameters such as total bilirubin, Child-Pugh grade, and albumin-bilirubin grade (*P* < 0.001 each). Moreover, considerably high correlations were observed between LSRi calculated using four time points and that calculated using three time points (*r* > 0.973 each), indicating that the timing bias of imaging was minimal.

**Conclusions:**

This study propose a novel time-associated radiological assessment, and revealed that the LSRi correlated significantly with traditional liver functional parameters. Changes in LSR over time may provide a superior preoperative assessment of regional liver function that is better for predicting post-hepatectomy liver failure than LSR using the hepatobiliary phase alone.

## Background

Liver resection is used for treating benign and malignant liver disease, and the incidence of post-hepatectomy liver failure (PHLF) has been reported within the range of 0.7–34% [1, 2]. PHLF thus remains a major concern and has been shown to be a key cause of hepatectomy-related mortality [3, 4]. Precise preoperative assessment of liver function is therefore important to minimize the risks associated with liver surgery.

Liver functional assessments such as indocyanine green (ICG) retention rate at 15 min (ICGR15), Child-Pugh grade, and albumin-bilirubin (ALBI) grade have been reported for predicting PHLF [5, 6]. However, these traditional liver functional assessments, obtained using data from blood tests and clinical evaluations, reflect liver function as a whole, but not future remnant liver function after hepatectomy. Several studies have indicated the usefulness of radiological imaging for assessing the function of future remnant liver functional assessment using radiological imaging [7, 8]. Gadolinium-ethoxybenzyl-diethylenetriamine pentaacetic acid (Gd-EOB-DTPA) is a liver-specific contrast medium, and magnetic resonance imaging (MRI) using Gd-EOB-DTPA (EOB-MRI) is widely used for detecting liver tumors with high spatial resolution [9]. Gd-EOB-DTPA is a contrast agent with approximately 50% hepatocyte uptake via the organic anion transporter protein, and with subsequent extraction into the bile duct [10]. The uptake and excretion of Gd-EOB-DTPA thus offers potential for quantifying liver function, and several studies have reported that a low signal intensity of liver parenchyma in the hepatobiliary phase reflects poor liver function [8, 11–13]. Gd-EOB-DTPA has therefore been expected to provide a useful modality for assessing regional liver function with high spatial resolution [8, 11–13].

The liver-to-spleen signal intensity ratio (LSR) is evaluated by EOB-MRI in the hepatobiliary phase and has been used as a marker of regional liver function [12, 13]. We have also reported LSR of the future remnant liver region as a reliable preoperative assessment of liver function useful for predicting PHLF [14]. However, single-center investigations of radiological liver functional assessment using EOB-MRI are not readily applicable to other institutions, because timings of the hepatobiliary phase differ from 15 to 20 min after Gd-EOB-DTPA injection, depending on the institution [14–16]. Other factors such as the dose bias of Gd-EOB-DTPA and protocol bias of MRI acquisitions are also limitations for universal application of radiological liver functional assessment [7, 14, 15, 17]. Federico et al. described the key disadvantage of liver functional assessment using EOB-MRI as the need for specific competence and that almost all previous studies were single-center investigations [7]. Preoperative liver functional assessment of ICGR15 is also affected by the dose of ICG and timing of blood sampling. ICGR15 should therefore be performed under accurate injection with an appropriate amount of ICG and accurate blood sampling at 15 min after ICG injection. On the other hand, the ICG disappearance rate (ICG-K), which is calculated from the serum ICG concentration at multiple time points, reflects changes in ICG concentration over time without the dose bias of ICG or the timing bias of blood sampling [18, 19]. We expected this quantitative evaluation, which analyzed information on infused compounds at multiple time points, would exclude limitations such as the timing bias from imaging and protocol bias from MRI acquisition settings, and would facilitate the development of widely available radiological assessments of liver function. The aim of this study was to develop a novel time-associated radiological liver functional assessment with minimal bias, and verify its correlation with traditional liver functional parameters.

## Methods

### Patients

Between January 2013 and December 2018, a total of 312 consecutive patients underwent pancreatectomy for pancreatic cancer at the National Cancer Center Hospital East, Chiba, Japan. Of these 312 patients, 33 were excluded from analyses due to being contraindicated for EOB-MRI (n = 28) or having undergone prior splenectomy (n = 5). The remaining 279 patients who underwent standardized EOB-MRI for detecting liver metastasis from pancreatic cancer before pancreatectomy were evaluated. Since we were investigating fundamental changes in LSR over time in normal liver, patients with diagnosed liver diseases such as hepatocellular carcinoma were not selected, but we investigated patients with pancreatic cancer.

### Image analysis of EOB-MRI

**Fig. 1:**
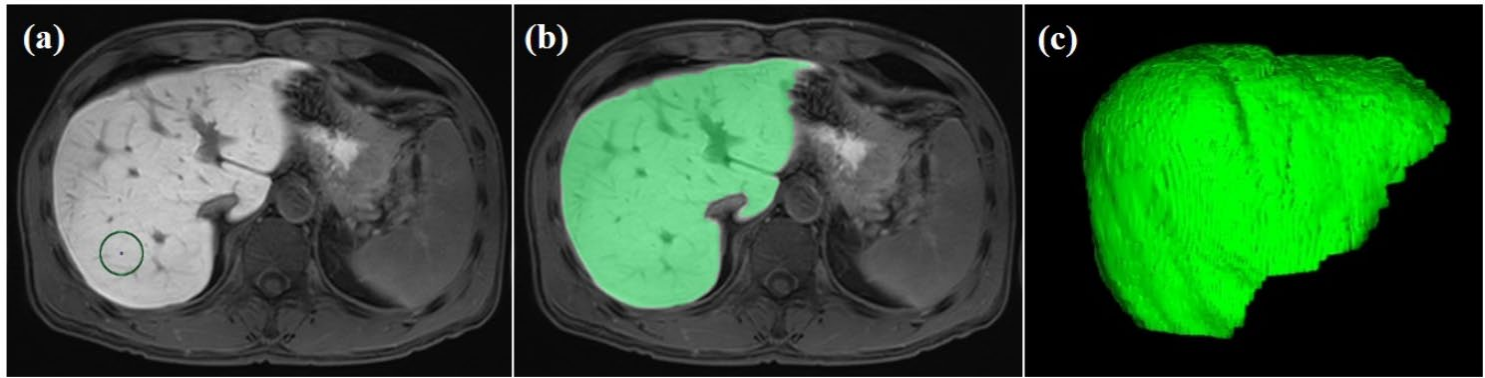
Image analysis using the three-dimensional volumetric system on magnetic resonance imaging with gadolinium-ethoxybenzyl-diethylenetriamine pentaacetic acid. (**a**) The investigator places a small volume of interest in liver parenchyma. (**b**) Liver parenchyma is semi-automatically extracted from the initial volume-of-interest seed. (**c**) The three-dimensional whole-liver volume is extracted, and the average whole-liver signal intensity is calculated automatically

LSR was calculated using a three-dimensional (3D) volumetric analysis system (SYNAPSE VINCENT; Fujifilm Medical, Tokyo, Japan) by a single investigator (M.K.) under the supervision of an experienced radiologist (T.K.). Figure 1 shows image analyses before pancreatectomy for the detection of liver metastasis from pancreatic cancer. The indicated liver parenchyma was semi-automatically extracted from a small, operator-defined volume of interest using the image-processing algorithm (Fig. 1a, b). The 3D whole-liver volume was then extracted (Fig. 1c), and the average whole-liver signal intensity was calculated automatically. Similarly, average whole-spleen signal intensity could be calculated. LSR was then calculated as the average signal intensity of the whole liver parenchyma divided by that of the whole splenic parenchyma. The LSRs of all patients were calculated at the following time points: pre-contrast phase, and 20 s, 1 min, 3 min, 10 min, and 15 min after intravenous administration of Gd-EOB-DTPA. The relationship between LSR and time after injection was then semi-logarithmically converted to be straightened, and LSRs at the four time points (1, 3, 10, and 15 min after injection) were plotted on a semi-logarithmic graph using a non-logarithmic scale for LSR (*y*-axis) and a logarithmic scale for minutes after infection (*x*-axis). Next, the approximate line of these four time points was created using the least-squares method. Finally, we defined the slope of the approximate line as the novel time-associated liver functional assessment termed “LSR increasing rate” (LSRi). We then investigated the correlation between LSRi and traditional liver functional parameters such as Child-Pugh grade and ALBI grade. Finally, we evaluated the timing bias of imaging for calculating LSRi by investigating the correlation between LSRi calculated by four time points and LSRi calculated by three time points.

### Clinical information

To investigate the relationship between LSRi and clinical factors, the following information at the time of MRI evaluation was collected from the medical records of patients: age, sex, medical history, blood tests, MRI equipment and acquisition protocol. The Child-Pugh score was calculated based on the following five variables: serum bilirubin level, serum albumin level, prothrombin activity, ascites status, and degree of encephalopathy. ALBI grade was calculated according to the following equation: -0.085 × (serum albumin level, g/L) + 0.66 × log (serum total bilirubin level, µmol/L). ALBI score was then categorized into the following three grades: ALBI 1, ≤ -2.60; ALBI 2, > -2.06 to ≤ -1.39; and ALBI 3, > -1.39.

### MRI protocol

All MRI studies were performed using 3.0-T scanners at our institute (Achieva or Ingenia; Philips Medical Systems, Amsterdam, the Netherlands). Contrast-enhanced 3D, fat-suppressed, T1-weighted images were obtained at 20 s, 1 min, 3 min, 10 min, and 15 min after intravenous administration of Gd-EOB-DTPA, using either of the following settings: repetition time 4 ms, echo time 2 ms, flip angle 10°, slice thickness 4.6 mm, and matrix size 512 × 512 for Achieva; or repetition time 3 ms, echo time 2 ms, flip angle 10°, slice thickness 4.6 mm, and matrix size 480 × 480 for Ingenia. Gd-EOB-DTPA was administered at a dose of approximately 0.1 mL/kg body weight, by rapid intravenous bolus injection using a power injector (Sonic Shot GX; Nemoto Kyorindo Co., Tokyo, Japan), at a rate of 2 mL/s.

### Statistical analysis

Continuous variables are presented as the median and range. The slope formula is the vertical change in median LSR on the y-axis divided by time (minutes) after contrast medium injection on the x-axis. Differences in median LSR among patients with or without hyperbilirubinemia were evaluated using pairwise comparisons with the Mann-Whitney test. Relationships between LSRi and clinical factors were also evaluated using the Mann-Whitney test. Correlations between the LSRi calculated using four time points and the LSRi calculated using three time points were assessed using the standard Pearson’s correlation coefficient. Two-sided *P*-values of less than 0.05 were considered indicative of significance. Statistical analysis was performed using JMP version 12.0.10 software (SAS Institute, Cary, NC).

## Results

### Change in LSR over time after Gd-EOB-DTPA injection

**Fig. 2:**
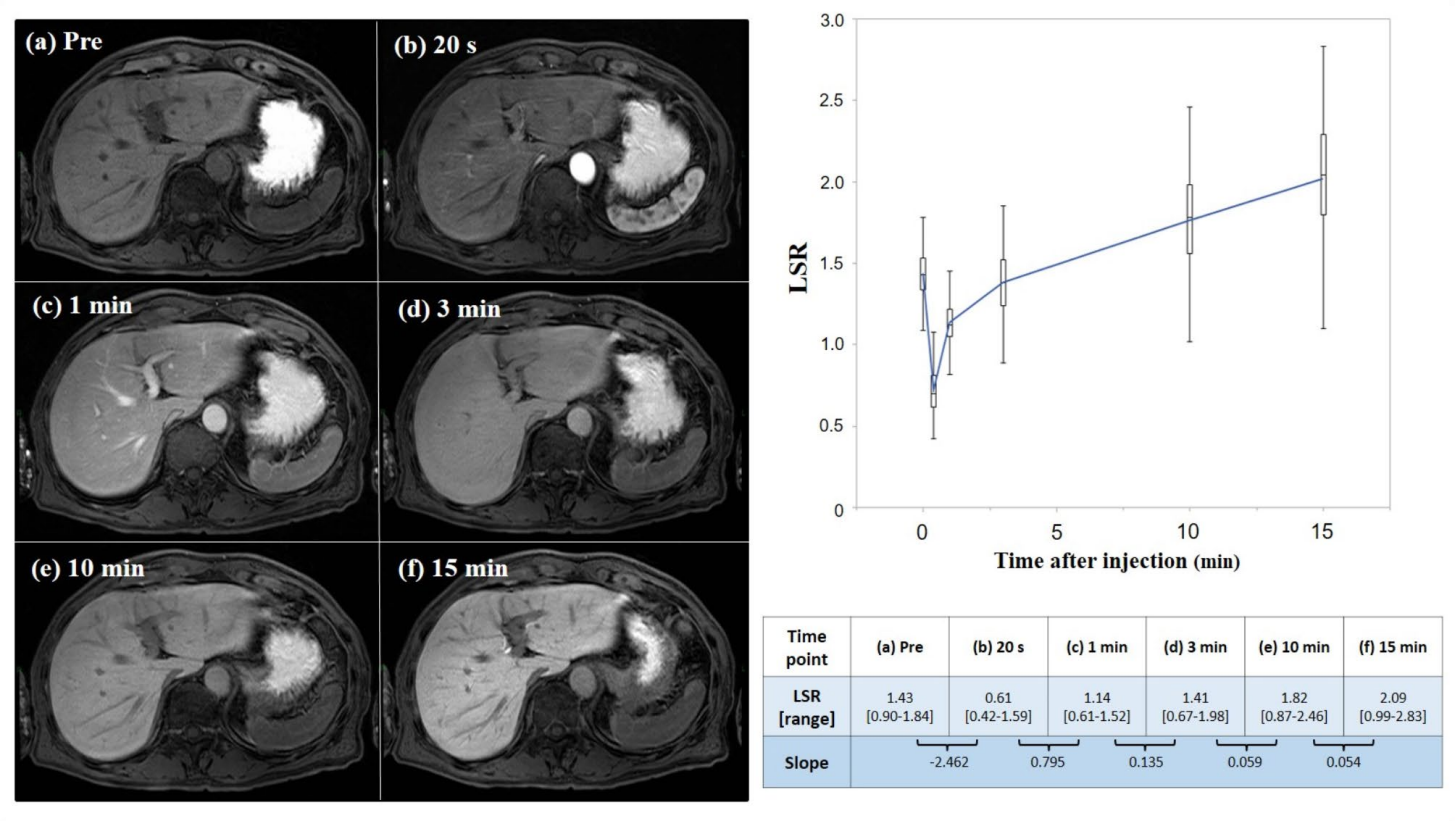
Time-associated change in liver-to-spleen signal intensity ratio (LSR). (**a**) Median LSR in the pre-contrast phase is 1.43 (range, 0.90–1.84). (**b**) After contrast medium injection, the increase in spleen signal intensity immediately decreases the LSR. (**c**) One minute after contrast medium injection, the signal intensity of liver parenchyma is similar to that of spleen parenchyma, representing the equilibrium phase. (**d**–**f**) After the equilibrium phase, LSR continuously increases but the slope gradually decreases

Figure 2 shows changes in LSR before and after Gd-EOB-DTPA injection. Median LSR in the pre-contrast phase was 1.43 (range, 0.90–1.84) (Fig. 2a). After Gd-EOB-DTPA injection, the rapid increase in spleen signal intensity resulted in a decrease in LSR (Fig. 2b). One minute after Gd-EOB-DTPA injection, liver signal intensity was increased and median LSR in the equilibrium phase (1 min after contrast medium injection) was 1.14 (range, 0.61–1.52) (Fig. 2c). The slope of the linear function from 20 s to 1 min after Gd-EOB-DTPA injection was 0.795. After the equilibrium phase, LSR continuously increased, but the slope gradually decreased, so a sequential change in LSR created a quadratic curve (Fig. 2d-f).

### Difference in LSR difference according to serum bilirubin

**Fig. 3:**
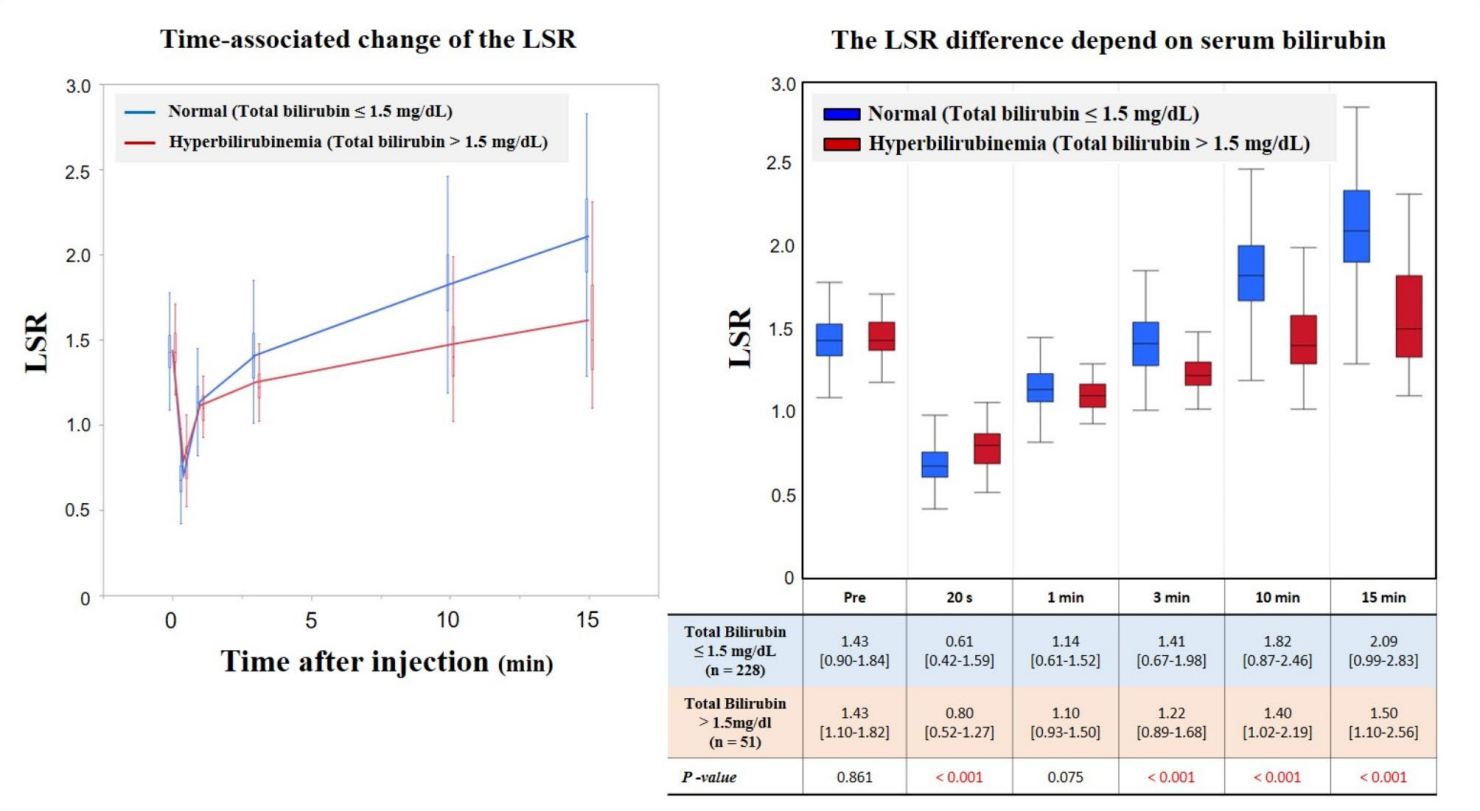
Time-associated changes in liver-to-spleen signal intensity ratio (LSR) according to serum bilirubin level. Median LSR in the pre-contrast phase does not differ significantly between groups. Twenty seconds after contrast medium injection, LSR is significantly lower in normal patients than in patients with hyperbilirubinemia. One minute after contrast medium injection, LSR in normal patients is equivalent to that in patients with hyperbilirubinemia. Subsequently, LSR is significantly higher in normal patients than in patients with hyperbilirubinemia, and the difference in LSR gradually increases over time

Figure 3 shows the time-associated change in the LSR based on serum bilirubin level. From the 279 patients studied, 51 patients were diagnosed with hyperbilirubinemia (total bilirubin > 1.5 mg/dL) due to obstructive jaundice at the time of MRI examination. In the pre-contrast phase, median LSR did not differ significantly between the presence or absence of hyperbilirubinemia (1.43 each, *P* = 0.861). Twenty seconds after Gd-EOB-DTPA injection, median LSR was significantly higher in patients with hyperbilirubinemia (0.80) than in patients without hyperbilirubinemia (0.61, *P* < 0.001). One minute after injection, median LSR in normal patients (1.14) was comparable to that in hyperbilirubinemia patients (1.10, *P* = 0.075). After the equilibrium phase, median LSR was significantly higher in normal patients than in patients with hyperbilirubinemia, and differences in LSR gradually increased over time.

### Liver functional assessment using changes in LSR over time

**Fig. 4:**
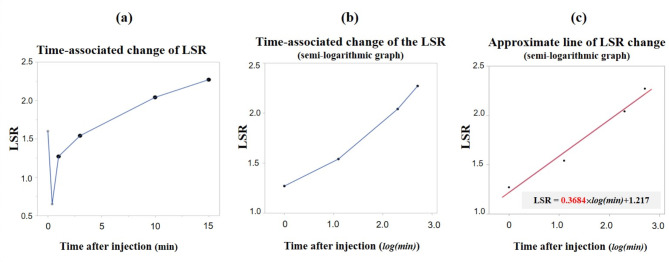
The definition of liver-to-spleen signal intensity ratio (LSR) increasing rate (LSRi). (**a**) Dynamic information on the LSR. Values of LSR calculated using four time points (1, 3, 10, and 15 min after injection) are selected. (**b**) Information from these four time points is plotted on a semi-logarithmic graph using a non-logarithmic scale for LSR (*y* axis) and a logarithmic scale for time after infection (*x* axis). (**c**) The approximate line of information from these four time points is created using the least-squares method, and the slope of the approximate line is defined as LSRi. The LSRi in this case is 0.3684

We determined the quantitative value of the change in LSR over time as follows. First, LSRs at four time points (1, 3, 10, and 15 min after contrast medium injection) were selected (Fig. 4a). We excluded the early phase (20 s after Gd-EOB-DTPA injection) because of the unstable hemodynamics of the liver and spleen parenchyma. We considered that the LSRi would represent only changes in liver signal intensity, as a previous study reported that the contrast effect of spleen enhancement rapidly returned to baseline by 1 min after injection [20]. The relationship between LSR and time after injection was then semi-logarithmically converted to be straightened, and LSRs at the four time points (1, 3, 10, and 15 min after injection) were plotted on a semi-logarithmic graph using a non-logarithmic scale for LSR (*y*-axis) and a logarithmic scale for minutes after infection (*x*-axis) (Fig. 4b). Next, the approximate line of these four time points was created by the least-squares method, and the slope of the approximate line was defined as the LSRi (Fig. 4c). This quantitative analysis using information from multiple time points is similar to the methodology for calculating ICG-K [21, 22].

### Visual assessment of relationships between changes in LSR over time and traditional liver function

**Fig. 5:**
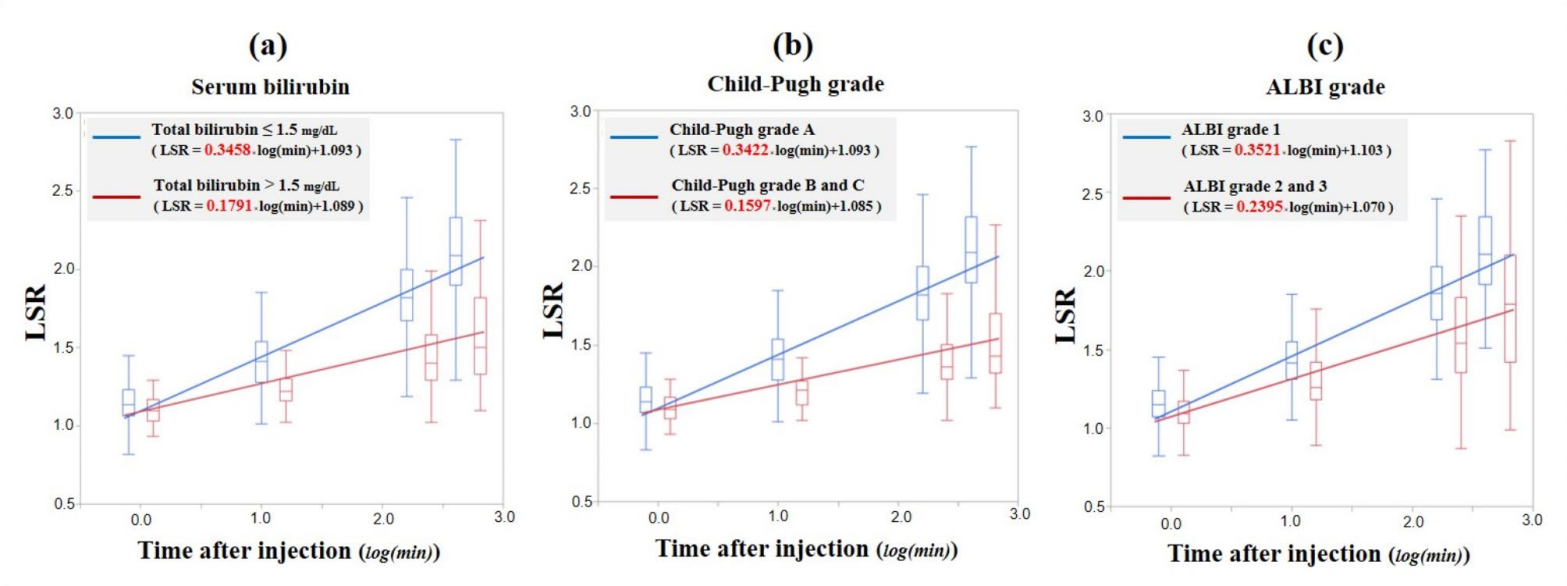
Approximate line calculated for median liver-to-spleen signal intensity ratio (LSR). (**a**) The slope of the approximate line is higher in patients without hyperbilirubinemia (slope, 0.3458) than in patients with hyperbilirubinemia (slope, 0.1791). (**b**) The slope of the approximate line is higher in patients with Child-Pugh grade A (slope, 0.3422) than in patients with Child-Pugh grade B or C (slope, 0.1597). (**c**) The slope of the approximate line is higher in patients with albumin-bilirubin (ALBI) grade 1 (slope, 0.3521) than in patients with ALBI grade 2 or 3 (slope, 0.2395)

For visual assessment of the relationship between the changes in LSR over time and traditional liver functional parameters, we compared the approximate line calculated by the median LSR in patient cohorts divided by traditional liver functional parameters (Fig. 5).

Figure 5a shows the approximate line depending on patient cohort divided by serum bilirubin level. The slope of the approximate line was higher in patients without hyperbilirubinemia (slope, 0.3458) than in patients with hyperbilirubinemia (slope, 0.1791). Similarly, slopes of the approximate line in patients with Child-Pugh grade A (Fig. 5b) and ALBI grade 1 (Fig. 5c) were higher than in patients with poor liver function.

### Correlations between LSRi and traditional liver functional assessments

Correlations between LSRi and clinical factors are summarized in Table 1. In all 279 patients, the following liver diseases were included: hyperbilirubinemia due to obstructive jaundice (n = 51); fatty liver (n = 28); and viral hepatitis (n = 12). Patient factors including traditional liver functional assessments are presented as median and range. Significant correlations were observed between LSRi and the following parameters: fatty liver; Child-Pugh grade; ALBI grade; albumin; total bilirubin; and prothrombin time (*P* < 0.001 each). Conversely, the differences in MRI equipment and slice thickness did not significantly affect the value of LSRi.

**Table 1:** Correlations between liver-to-spleen signal intensity ratio increasing rate (LSRi) and clinical factors

			LSR (15 min)	*P*		LSRi	*P*
**Age (years)**	≥ 70 (n = 146)		2.05 [1.10–2.83]	0.414		0.327 [0.049–0.517]	0.847
	< 70 (n = 133)		2.03 [0.99–2.77]			0.326 [0.060–0.566]	
**Sex**	Male (n = 156)		2.08 [1.10–2.83]	0.700		0.329 [0.049–0.544]	0.983
	Female (n = 123)		2.03 [0.99–2.77]			0.324 [0.059–0.566]	
**Hypertension**	Yes (n = 126)		1.99 [0.99–2.83]	0.074		0.319 [0.049–0.513]	0.093
	No (n = 153)		2.08 [1.19–2.77]			0.334 [0.052–0.566]	
**Diabetes**	Yes (n = 95)		2.00 [0.99–2.83]	0.387		0.319 [0.059–0.517]	0.209
	No (n = 184)		2.08 [1.19–2.77]			0.332 [0.049–0.566]	
**Dyslipidemia**	Yes (n = 81)		2.04 [0.99–2.83]	0.739		0.329 [0.060–0.513]	0.571
	No (n = 198)		2.04 [1.11–2.77]			0.325 [0.049–0.566]	
**Viral hepatitis**	Yes (n = 12)		2.03 [1.53–2.60]	0.547		0.304 [0.195–0.499]	0.586
	No (n = 267)		2.05 [0.99–2.83]			0.327 [0.049–0.566]	
**Previous chemotherapy**	Yes (n = 39)		1.99 [1.48–2.83]	0.541		0.319 [0.168–0.513]	0.571
	No (n = 240)		2.05 [0.99–2.77]			0.329 [0.049–0.566]	
**Fatty liver**	Yes (n = 28)		1.76 [1.27–2.42]	< 0.001*		0.248 [0.049–0.467]	< 0.001*
	No (n = 251)		2.08 [0.99–2.83]			0.332 [0.052–0.566]	
**MRI equipment**	Achieva (n = 166)		2.06 [0.99–2.83]	0.198		0.331 [0.049–0.566]	0.267
	Ingenia (n = 113)		2.02 [1.11–2.76]			0.323 [0.052–0.544]	
**Slice thickness (mm)**	≥ 4.8 (n = 92)		1.99 [1.10–2.83]	0.539		0.324 [0.049–0.513]	0.639
	< 4.8 (n = 187)		2.05 [0.99–2.68]			0.330 [0.058–0.566]	
**Child-Pugh grade**	A (n = 238)		2.09 [0.99–2.77]	< 0.001*		0.341 [0.129–0.566]	< 0.001*
	B or C (n = 41)		1.43 [1.10–2.83]			0.114 [0.049–0.513]	
**ALBI grade**	1 (n = 188)		2.11 [1.51–2.77]	< 0.001*		0.355 [0.168–0.566]	< 0.001*
	2 or 3 (n = 91)		1.79 [0.99–2.83]			0.254 [0.049–0.513]	
**Platelet count (×10** ^**4**^ **/µL)**	≥ 15.8 (n = 245)		2.04 [1.10–2.83]	0.360		0.324 [0.049–0.566]	0.102
	< 15.8 (n = 34)		2.06 [0.99–2.76]			0.356 [0.138–0.512]	
**Albumin (g/dL)**	≥ 4.1 (n = 180)		2.09 [1.30–2.77]	< 0.001*		0.352 [0.074–0.566]	< 0.001*
	< 4.1 (n = 99)		1.90 [0.99–2.83]			0.296 [0.049–0.513]	
**Total bilirubin (mg/dL)**	≥ 1.5 (n = 51)		1.50 [1.10–2.56]	< 0.001*		0.150 [0.049–0.451]	< 0.001*
	< 1.5 (n = 228)		2.09 [0.99–2.83]			0.345 [0.129–0.566]	
**Prothrombin time (%)**	≥ 70 (n = 268)		2.06 [1.10–2.83]	< 0.001*		0.329 [0.052–0.566]	< 0.001*
	< 70 (n = 11)		1.44 [0.99–2.09]			0.138 [0.049–0.385]	

ALBI grade, albumin-bilirubin grade; LSR, liver-to-spleen signal intensity ratio; LSRi, liver-to-spleen signal intensity ratio increasing rate; MRI, magnetic resonance imaging

* *P* < 0.05; continuous variables were analyzed using the Mann-Whitney test, and are presented as median and range

### Timing bias of imaging for calculating LSRi

**Fig. 6:**
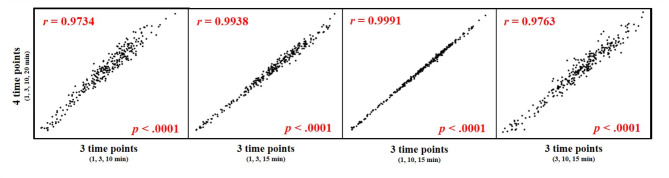
Variabilities between liver-to-spleen signal intensity ratio increasing rate (LSRi) as calculated using four time points and LSRi as calculated using three time points. Considerably high correlations are evident between LSRi using four time points and LSRi using three time points (*r* > 0.973 each)

In the present study, we initially calculated LSRi using information from four time points (1, 3, 10, and 15 min). To investigate the timing bias of imaging for calculating LSRi, we also calculated LSRi using three time points, and compared the values of LSRi calculated using four time points and those calculated using three time points. Figure 6 shows the variabilities in LSRi calculated by four time points and LSRi calculated by three time points. Considerably high correlations were observed between LSRi values calculated by each method (*r* > 0.973 each).

## Discussion

This study focused on sequential changes in liver signal intensity of EOB-MRI and developed LSRi as a novel, time-associated radiological assessment of liver function. LSRi showed significant correlations with traditional liver functional parameters such as Child-Pugh grade, ALBI grade, albumin, total bilirubin, and prothrombin time. Moreover, the present study revealed that potential limitations, such as timing bias in imaging, the difference in MRI equipment, and the difference in slice thickness, had little impact on LSRi.

Liver functional assessments can be divided in two groups: passive (non-time-associated); and dynamic (time-associated). Passive assessments of liver function include evaluations of albumin, bilirubin, and prothrombin activity. Systems for scoring liver function such as Child-Pugh grade and ALBI grade are also passive assessments of liver function. Previous reports have suggested that these types of passive assessments cannot be used as rapid indicators of changes in liver function, and are not accurate enough to predict outcomes after liver surgery [23, 24]. In contrast, dynamic quantitative liver function tests such as ICG-K, which are calculated from ICG concentrations at multiple time points, allow precise assessment of liver function by time-associated uptake or the metabolic capacity for infused compounds. Previous basic and clinical reports have suggested that dynamic quantitative liver function tests provide more accurate assessment of the specific aspects of liver function than passive assessments [23–25]. Some researchers have classified ICGR15 as a dynamic liver function test, but ICGR15 does not strictly reflect time-associated changes in the concentration of ICG, because evaluation is only made from two time points, before and 15 min after injection [18, 23–25]. Yokoyama et al. reported preoperative liver functional assessment using ICG-K as a more useful parameter for predicting PHLF than ICGR15 [25]. The third edition of clinical practice guidelines for the management of biliary tract cancers published by the Japanese Society of Hepato-Biliary-Pancreatic Surgery also recommend using the ICG-K for preoperative assessment of liver function [26]. However, to the best of our knowledge, no previous reports have investigated radiological assessments of liver function over time. Changes in LSR over time may provide a superior preoperative assessment of regional liver function for predicting PHLF than LSR using the hepatobiliary phase alone. Investigation of preoperative liver function using the LSRi to predict PHLF in patients undergoing major hepatectomy remains a matter for future research.

The investigation of radiological liver functional assessment using EOB-MRI has several limitations, such as timing bias in imaging, dose bias for Gd-EOB-DTPA, and protocol bias for MRI acquisitions [14–17]. Preoperative assessment of liver function by ICGR15 similarly involves timing bias in blood sampling and should be evaluated accurately at 15 min after ICG injection. Conversely, ICG-K, which is calculated by multiple blood samplings after injection, reflects changes in ICG concentration over time and is known to provide functional assessment of the liver with minimal timing bias for blood sampling [18]. Kumasawa et al. reported that ICG-K calculated using blood samples at 3-min intervals after injection correlated closely with results calculated from values at 2-min intervals [18]. The present study revealed high correlations between LSRi calculated using four time points and LSRi calculated using three time points. These results show that LSRi calculated using EOB-MRI information from multiple time points has minimal timing bias.

Liver functional assessment using EOB-MRI in the hepatobiliary phase is affected not only by timing bias, but also by dose bias of contrast medium and protocol bias of MRI acquisitions. Similarly, ICGR15 shows a dose bias and requires accurate injection with an appropriate amount of ICG for the weight of the patient [19]. In contrast, ICG-K has been used for liver functional assessment with minimal dose bias for ICG [19]. Aono et al. reported that the ICG-K with a 0.5-mg/dL dose of ICG was almost the same as that with a 0.1-mg/dL dose [19]. This report suggests the speed at which ICG disappears from plasma, as the change in ICG over time, offers a universal quantitative assessment regardless of the ICG dose. Considering the similar metabolic pathways for ICG and Gd-EOB-DTPA, the LSRi reflecting changes in Gd-EOB-DTPA uptake over time is expected to provide a universal quantitative assessment of liver function regardless of Gd-EOB-DTPA dose. Moreover, the present study suggested that protocol biases in MRI acquisition such as MRI equipment and slice thickness do not significantly affect the value of the LSRi (Table 1). LSRi is therefore expected to facilitate radiological assessment of liver function in multiple centers regardless of protocol bias in MRI acquisitions.

A previous report suggested that ICGR15 and ICG-K values would be worsened with obstructive jaundice, but would recover after biliary drainage [27]. Clinical practice guidelines therefore recommend that ICGR15 and ICG-K for preoperative liver function before hepatectomy should be performed after obtaining relief from obstructive jaundice [26]. Because hyperbilirubinemia worsened LSRi in the present study, supplementary investigations are necessary to clarify whether LSRi in patients with obstructive jaundice recovers after biliary drainage. Of the 279 patients in the present study, 228 showed normal serum bilirubin at the time of MRI evaluation. Among those 228 patients, 47 had a history of biliary drainage before MRI evaluation, and the remaining 181 had no history of biliary drainage or hyperbilirubinemia during the preoperative clinical course. We compared LSRi values between the 181 normal patients and the 47 patients who recovered from obstructive jaundice by biliary drainage before MRI evaluation. LSRi in patients with a history of biliary drainage (0.327) did not differ significantly from that in normal patients (0.355, P = 0.364). This result suggests that LSRi after relief from obstructive jaundice may prove useful for preoperative assessment of liver function, similar to ICGR15 and ICG-K. Future studies investigating the association between LSRi and PHLF should also focus on patients who recovered from hyperbilirubinemia due to obstructive jaundice.

Our study had several limitations. First, this was a retrospective, single-center, cohort study of pancreatic cancer patients, so our liver functional assessments should be validated in other institutions before clinical application as a tool for assessment of liver function using EOB-MRI in multiple centers. Moreover, this retrospective study did not include patients diagnosed with background liver diseases such as liver cirrhosis, liver steatosis, or liver fibrosis. The Child-Pugh grade and ALBI grade are also liver functional evaluation originally used for chronic liver injury such as viral hepatitis and non-alcoholic liver disease. The correlations between LSRi and background liver diseases should therefore be investigated. Relationships among the LSRi, ICG, ICG-K, and clinical outcomes such as PHLF are also worthwhile subjects for future study. To clarify the superiority of the LSRi compared to LSR, ICG, and ICG-K as preoperative liver functional assessments, investigation of the impact of the LSRi on PHLF is necessary. Second, results for regional liver function, as the advantage of liver functional assessment using radiological imaging, were not investigated in the present study. The LSRi of the future remnant liver region could be calculated by 3D volumetric analysis. Preoperative evaluation of regional liver function using LSRi for hepatectomy should be examined in a future study. Third, the 3D volumetric analysis system sometimes automatically extracts extrahepatic-parenchymal tissues such as portal veins, hepatic veins, small cysts and tumors. However, our previous report showed a high correlation between LSR and vascular subtraction LSR (LSR excluding extrahepatic-parenchymal tissues), and demonstrated that the LSR adequately reflects contrast enhancement of the liver parenchyma [8]. Our previous report suggested that liver function can be evaluated without subtracting vessels and vascular perfusion areas. Finally, several protocol biases such as repetition time, echo time, flip angle, magnetic fields strength, and doses of contrast medium were not investigated in the present study, and differences among MRI manufacturers (such as SIEMENS, GE, and TOSHIBA) were not evaluated. These limitations should be investigated in further multicenter research.

## Conclusions

The present study revealed that the LSRi, calculated as the change in LSR over time, correlated significantly with traditional liver functional parameters. This novel time-associated information on EOB-MRI may overcome the limitations of radiological assessment of liver function, and is expected to become widely available as a preoperative liver function parameter.

## Data Availability

The datasets generated and/or analyzed during the present study are available from the corresponding author upon reasonable request.

## Abbreviations

ALBI: Albumin-bilirubin
EOB-MRI: Magnetic resonance imaging using gadolinium-ethoxybenzyl-diethylenetriamine pentaacetic acid
Gd-EOB-DTPA: Gadolinium-ethoxybenzyl-diethylenetriamine pentaacetic acid
ICG: Indocyanine green
ICGR15: Indocyanine green retention rate at 15 min
LSR: Liver-to-spleen signal intensity ratio
LSRi: Liver-to-spleen signal intensity ratio increasing rate
MRI: Magnetic resonance imaging
PHLF: Post-hepatectomy liver failure
